# Ochratoxin A reduction ability of biocontrol agent *Bacillus subtilis* isolated from Korean traditional fermented food Kimchi

**DOI:** 10.1038/s41598-018-26162-5

**Published:** 2018-05-23

**Authors:** Shruti Shukla, Jung Hyun Park, Soo Hyun Chung, Myunghee Kim

**Affiliations:** 1 Department of Food Science and Technology, Yeungnam University, Gyeongsan-si, Gyeongsangbuk-do 38541 Republic of Korea; 2 Department of Energy and Materials Engineering, Dongguk University-Seoul, Seoul, 04620 Republic of Korea; 3 Department of Integrated Biomedical and Life Science, Korea University, Seoul, 02841 Republic of Korea

## Abstract

In the present study, a new biocontrol strain, *Bacillus subtilis* KU-153, was isolated from the Korean traditional fermented food Kimchi and evaluated for its ability to reduce the ochratoxin A (OTA) content in culture medium. A 16 S rRNA gene sequencing analysis revealed the identity of newly isolated strain KU-153 as *B. subtilis*. The growth kinetic study of *B. subtilis* KU-153, in terms of the OTA reduction in culture medium, confirmed its biocontrol efficacy. To verify its ability to reduce the OTA content in culture medium, bacterial extracts (intracellular and extracellular) of *B. subtilis* were separated and compared with whole *B. subtilis* cells (viable and heat-killed). No reduction in the OTA content was observed in culture medium with extracellular and intracellular extracts, while viable and heat-killed cells of *B. subtilis* showed significant levels (*p* < 0.05) of OTA reduction in culture medium. Interestingly, *B. subtilis* heat-treated cells showed a higher OTA reduction (45%) than viable cells (22%). Further, *B. subtilis* heat-treated cells were assessed for their ability to reduce OTA levels in artificially contaminated red wine samples that resulted in an OTA reduction of approximately 90%, suggesting the biocontrol potential of the newly isolated strain *B. subtilis* KU-153 on OTA reduction.

## Introduction

Ochratoxins are a group of mycotoxins produced mainly by the strains of some *Aspergillus* and *Penicillium* species. The family of ochratoxins consists of three members, A, B, and C, which differ slightly from each other in their chemical structures. These differences, however, have marked effects on their respective toxic potentials. Various toxic effects of ochratoxin A (OTA) have been reported, such as teratogenic, mutagenic, nephrotoxic, hepatotoxic, immunotoxic, and carcinogenic effects. These are due to the inhibition of protein synthesis, promotion of membrane lipid peroxidation, disruption of calcium homeostasis, and DNA damage^1^.

Owing to the harmful effects of OTA and an increasing knowledge of health hazards, many countries have established a limit for OTA in food and feed. At the 37^th^, 44^th^, and 56^th^ meetings of the Joint FAO/WHO Expert Committee on Food Additives (JECFA), a provisional tolerable weekly intake of 100 ng/kg body weight for OTA was established^2^. In a recent proposal from the European Union, which has been effective since October 1, 2006, the maximum tolerated limit for OTA was reduced to below 5 ng/kg body weight/day^3–5^. According to the European Commission (Regulation 1881/2006), the maximum contamination level of OTA in processed cereal-based foods and baby foods for infants and young children and in dietary foods for special medical purposes intended specifically for infants is 0.5 ng/g, while that for unprocessed cereals and coffee is 5 ng/g^3^. Finally, based on the available scientific toxicological and exposure data, the European Commission has produced a set of legal limits for OTA in different food products: 5 µg/kg, 10 µg/kg, 2 µg/kg, and 0.5 µg/kg for grains and grain products, instant coffee, grape juice and wine, and infant formula, respectively^6,7^.

Based on the harmful effects of OTA on human health and the economic losses caused by food and feed contamination, it is necessary to reduce the risk of exposure to these compounds, mostly through preventive measures and treatments. Innovative technologies have been suggested to reduce the amount of OTA in food and feed^8^. The use of synthetic chemicals and fungicides has cumulative and residual effects and is harmful to human life and development^9^. Biological control using antagonistic microorganisms has long been proposed as a good option for controlling plant pathogens^10^. One of the advantages of biocontrol is that it could be used together with fungicides, reducing their hazardous effects and helping to inhibit fungal growth^11,12^. A variety of different microbial species have been reported as biocontrol agents for various food products associated with pathogen and toxin development^13–15^. Under these situations, biological control methods could be promising and safe alternatives for reducing the OTA contents of foods by acting against ochratoxigenic fungal species without causing harmful effects to humans or the environment. During the selection of microbial strains for biocontrol agents, the selected strain must be Generally Recognized As Safe (GRAS)^16^.

Kimchi is a well-known Korean traditional fermented vegetable food that is generally considered to be one of the five healthiest foods in the world^17^. Kimchi fermentation is typically characterized by the presence of various microorganisms such as *Bacillus subtilis* and *Lactobacillus* species, which are considered safe for human consumption based on their GRAS status. The United States of Food and Drug Administration has also recognized some substances derived from *B. subtilis* (from fermented foods) as GRAS, and this species is also used as a probiotic^18^. *B. subtilis* is considered to be an important biocontrol agent against several toxin-producing pathogenic fungal strains^19^. Several studies also confirm that *Bacillus* species, which are widely used in the food industry, have exceptional abilities to eliminate various other toxins^20^. Although yeasts and several other lactic acid bacteria have shown promising biocontrol potentials against food toxins^21^, there have been only a few reports regarding their abilities to reduce OTA in food and feed. Therefore, there is an urgent need to identify natural and safe biocontrol agents to control various mycotoxins, such as OTA. In the present study, we isolated a bacterial strain, *B. subtilis* KU-153, from the Korean traditional fermented food Kimchi and confirmed its ability to reduce the OTA contents in culture media, leading to establish a hypothesis of its mechanistic role in OTA reduction.

## Materials and Methods

### Chemicals and reagents

OTA standard solution (10 µg/mL in acetonitrile) and OTA standard powder (1 mg) were purchased from Sigma-Aldrich (St. Louis, MO, USA) and stored at −20 °C. Ochratoxin alpha (OTα) standard solution (10 µg/mL in acetonitrile) was purchased from LGC Standards (Wesel, North Rhine-Westphalia, Germany) and stored at −4 °C. Nutrient broth (NB), nutrient agar (NA), and 1% skim milk were purchased from Becton, Dickinson and Company (Sparks, MD, USA) and used in the enrichment culture and isolation culture. Ethyl acetate (Fisher Scientific Korea; Seoul, Korea), acetonitrile (Merck, Darmstadt, Germany), acetic acid (Avamtor Performance Material Inc.; Center Valley, PA, USA), and methanol (Sigma-Aldrich; St. Louis, MO, USA) were of high-performance liquid chromatography (HPLC) grade. Water for HPLC was purified with a water purification system (Duplex 250 H, Lucky Scientech Co. Ltd., Bucheon, Korea). A silica gel plate (Silica gel 60 without fluorescent indicator; Merck, Darmstandt, Germany), 0.2 μm syringe filter (Whatman, GE Healthcare; Kent, England), and formic acid (Yakuri Chemicals; Osaka, Japan) were purchased and used in subsequent experimental analyses.

### Bacterial strains

Reference strains of *B. subtilis* KCTC 13021, KCTC 13241, KCTC 3014, KCTC 13112, KCTC 13429, and KCTC 1666 were obtained from the Korean Culture Type Collection (Daejeon, Korea) and were grown in NB at 35 °C for 24 h and stored as a stock culture at −20 °C until further use.

### Isolation of bacterial strains from Kimchi

Bacterial colonies were isolated from the Korean traditional fermented food Kimchi. Briefly, Kimchi broth was appropriately diluted using a sterile 0.85% NaCl solution, and then the diluted samples were spread on NA plates supplemented with 1% skim milk to isolate protease-producing *Bacillus* species^22^. Proteolytic activities of *Bacillus* spp. were detected on the basis of appearance of clear zones around the bacterial colonies. The protease positive *Bacillus* colonies were picked up and used in further studies for analyzing their potential to reduce OTA. These colonies were sub-cultured on new NA plates (with or without skim milk) followed by incubation at 35 °C for 24 h, and further stock cultures were prepared and stored at −20 °C.

### Screening for bacterial strains capable of reducing OTA content

**Figure 1:**
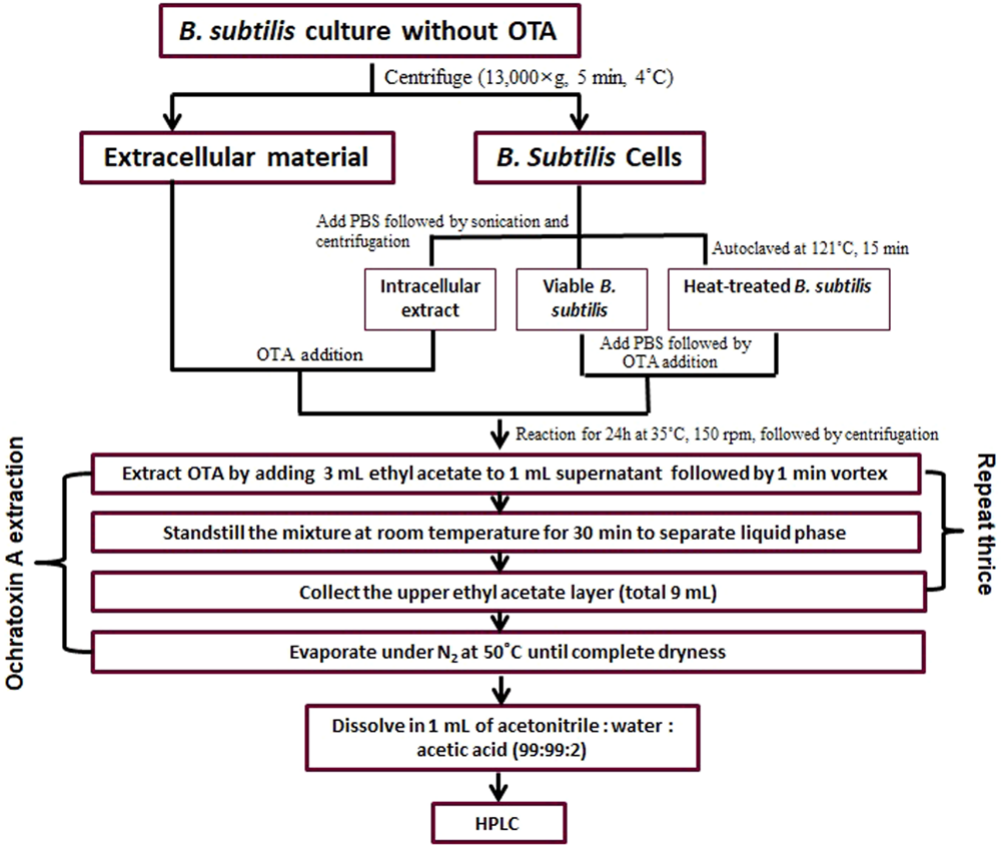
Procedure for screening bacterial strains capable of reducing ochratoxin A.

To analyze the OTA reduction abilities of the all the grown protease positive *Bacillus* spp. (10 colonies), isolates were inoculated in 25 mL of NB containing 40 µg/L OTA and incubated at 35 °C and 150 rpm in the dark for 48 h using a shaking incubator (HB-201SF, Hanbaek Scientific Co.; Bucheon, Korea). Cells were removed from NB by centrifugation (13,000 × *g*, 5 min, 4 °C) using a centrifuge (Combi-514R, Hanil Science Industrial, Gangneung, Korea). OTA was extracted from 2 mL of bacterial supernatant by mixing with 3 mL of ethyl acetate, and this procedure was repeated thrice. The total organic phase (9 mL) was evaporated to dryness at 50 °C using a nitrogen evaporator (MD 200-1, Allsheng Limited; Zhejiang, China) and further dissolved in 50 µL of methanol. A detailed schematic for the OTA extraction protocol is presented in Fig. 1.

Additionally, a thin layer chromatographic (TLC) method for OTA analysis was carried out as described by Teren *et al*.^23^. The extracted solution (20 µL) was spotted onto silica gel plates (Silica gel 60 without fluorescence) using a mixture of toluene:ethyl acetate:90% formic acid (5:4:1, v/v) as a developing solvent. When the eluent reached 3/4 of the distance from the baseline of the plate, the TLC plate was removed from the developing tank, dried at room temperature, and illuminated under UV light (λ = 360 nm) in a dark room as a bluish-green fluorescent spot^24^. Purified OTA was spotted as a reference OTA standard.

### Identification of the selected bacterium

To identify the selected bacterial colony of the OTA-reducing bacterial strain, molecular identification was performed, involving DNA extraction using a DNeasy Plant Mini-Kit (Qiagen; Valencia, CA, USA) and subsequent DNA amplification of the 16 S rRNA gene. Single-pass sequencing of the amplified nucleotide product was performed on each template using the 785 F primer (5′-GGATTAGATACCCTGGTA-3′) and 907R′ primer (5′-CGTCAATTCMTTTRAGTTT-3′). The standard PCR conditions were as follows: one cycle of denaturation at 95 °C for 15 min followed by 30 cycles of denaturation at 95 °C for 20 s, annealing at 50 °C for 40 s, extension at 70 °C for 1 min 30 s, and one cycle of extension at 70 °C for 5 min. PCR products were visualized by electrophoresis in 1% (w/v) agarose gel stained with ethidium bromide. Sequence search and analysis was performed using the standard nucleotide BLAST [National Center for Biotechnology Information (NCBI), Library of Medicine; Bethesda, MD, USA; http://www.ncbi.nlm.nih.gov/BLAST/] against GenBank. BLAST outputs were sorted based on maximum identity and query coverage. Hits that showed 97% or higher sequence identity were chosen as probable candidates for identification. A sequence that showed ≥97% identity with several species of the same genus was identified up to the genus level only, whereas a match of ≥99% identity with a single species resulted in identification of the strain at the species level as *B. subtilis*. The sequences of the 16 S rRNA gene of the *B. subtilis* isolate were submitted to the GenBank database under accession number KX950748. The isolated strain was also deposited in the Korean Culture Collection Center with the identification number KCCC 92146 P.

### Preparation of bacterial inoculum

A bacterial strain inoculum was obtained by growing *B. subtilis* in NB and incubating overnight at 35 °C prior to growth kinetics and OTA reduction studies.

#### B. subtilis growth kinetics and OTA reduction

To analyze the kinetics of the OTA reduction, *B. subtilis* was inoculated in 300 mL of NB containing 40 µg/L OTA and incubated at 35 °C and 150 rpm for 25 h in the dark. The culture broth was collected after every 0, 5, 10, 15, 20, and 25 h of incubation time. The cell count of the culture broth was measured by plating on agar plates and incubating the plates at 35 °C for 18–24 h for the assessment of bacterial growth rate and reduced OTA content in the culture broth. Subsequently, the culture broth was centrifuged, followed by OTA extraction.

### HPLC conditions and analysis

Quantitative analysis of OTA content was carried out by HPLC using a Dionex Ultimate 3000 UHPLC system (Thermo Scientific; Sunnyvale, CA, USA) equipped with a fluorescence detector (excitation wavelength = 330 nm, emission wavelength = 460 nm) and Capcell Pak C18 column (4.6 mm width × 250 mm length, 5 µm pore size, Shiseido; Tokyo, Japan). As a mobile phase, a solvent mixture of acetonitrile:water:acetic acid (99:99:2, v:v:v) was pumped at a flow rate of 0.8 mL/min, and the column temperature was maintained at 35 °C. OTA standard solutions of different concentrations (5, 10, 30, and 50 µg/L) and sample solutions of OTA extract were filtered through a 0.2 µm syringe filter prior to HPLC analysis, and 20 µL of each sample was injected for 20 min of run-time. Analysis of chromatograms was performed by comparisons with the standard curve of OTA.

### Comparison of OTA reduction abilities of extracellular fraction, intracellular fraction, and cells of *B. subtilis*

#### Preparation of extracellular fraction, intracellular fraction, and cells of B. subtilis

To analyze the OTA reduction activities of *B. subtilis* fractions and cells, both extracellular and intracellular fractions were first prepared with slightly modified methods^25–27^. Briefly, *B. subtilis* culture broth (10 mL) was inoculated into 600 mL of sterile NB, and *B. subtilis* was grown at 35 °C and 100 rpm for 48 h. The extracellular fraction was harvested by centrifugation (13000 × *g*, 5 min, 4 °C), and the cells were further used for isolation of the intracellular fraction. The harvested extracellular fraction was filtered through a 0.2 µm membrane filter (Sartorius; Goettingen, Germany).

The separated cell pellet was washed three times with 10 mL of 0.1 M PBS (pH 7.0) and then divided into two parts for the preparation of the intracellular fraction and cells. To prepare the intracellular fraction, 1 g of cells was suspended in 10 mL of 0.1 M phosphate buffer (pH 7.0) and then lysed for 5 min on ice using an ultrasonic cell disruptor (VC-750, Sonics and Materials, Inc.; Newtown, CT, USA). Cell disruption was typically performed by cell sonication at 35% amplitude for 5 s with a 15 s interval. The disintegrated cell suspension was centrifuged at 12,000 rpm for 5 min at 4 °C and filtered through a 0.2 µm syringe filter^27^. The remaining washed cell pellet was divided into two portions: one portion contained viable wet cells, and the other contained cells that were then heat-treated by autoclaving at 121 °C for 15 min.

#### OTA reduction assay

To analyze the OTA reduction capabilities of the extracellular and intracellular fractions of *B. subtilis* as well as of wet cells (viable and heat-treated), the extracellular (1.2 mL) and intracellular (1.2 mL) fractions as well as the viable and heat-treated wet cells were spiked with 40 µg/mL of OTA. The mixtures were reacted at 35 °C and 150 rpm for 24 h in the dark. Subsequently, the OTA was extracted from 1 mL of each mixture with ethyl acetate (Fig. 1) and dissolved in 1 mL of mobile phase (acetonitrile:water:acetic acid, 99:99:2, v/v/v) followed by filtration through a 0.2 µm syringe filter for HPLC analysis.

### Method validation

The analytical HPLC procedure was validated by means of calibration and evaluation of the range of linearity, limit of detection (LOD), limit of quantification (LOQ), and recovery rate. The calibration measurements were carried out with OTA standard solutions. The linear response of OTA was determined in the concentration range of 5–50 µg/L, which led to the correlation factor R^2^ > 0.99. LOD and LOQ were calculated using the equations LOD = X_0_ + 3 SD and LOQ = X_0_ + 5 SD, respectively, where X_0_ was the average response of the blank samples, and SD referred to the standard deviation for n = 6.

### Applicability in wine food matrix

In order to confirm the practical and industrial OTA reduction abilities of heat-treated *B. subtilis* cells, red wine was used as a food matrix. Briefly, 2 mL of red wine was spiked with 40 µg/mL of OTA, and then heat-treated cells of *B. subtilis* (0.4 g) were added to the mixture to confirm their ability to reduce OTA levels. The mixture was reacted at 35 °C and 150 rpm for 24 h in the dark. Subsequently, the OTA was extracted from 1 mL of mixture with ethyl acetate (Fig. 1) and dissolved in 1 mL of mobile phase (acetonitrile:water:acetic acid, 99:99:2, v/v/v) followed by filtration through a 0.2 µm syringe filter for HPLC analysis.

### Statistical analysis

All experiments were carried out in triplicate, and statistical analysis of the data was performed at a significance level of *p* < 0.05 using SPSS software (IBM SPSS Statistics 22, IBM Corp.; NY, USA). After analysis of variance, Duncan’s multiple range test was used for post-hoc analysis.

### Data availability

The authors declare that all the other data supporting the finding of this study are available within the article and from the corresponding author on reasonable request.

## Results and Discussion

### Screening and identification of OTA-reducing bacterial strains

**Figure 2:**
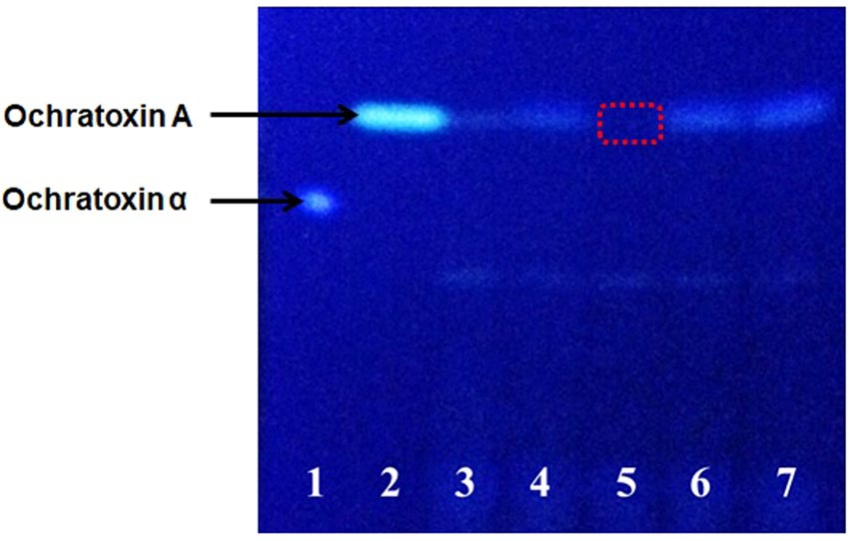
Thin layer chromatography of bacterial strains isolated from Kimchi and further grown in nutrient broth containing 40 µg/L OTA. 1: ochratoxin α (10 µg/mL), 2: ochratoxin A (10 µg/mL), 3: nutrient broth with 40 µg/L OTA, 4: Bacterial culture 1 spiked with 40 µg/L OTA, 5: Bacterial culture 2 spiked with 40 µg/L OTA, 6, Bacterial culture 3 spiked with 40 µg/L OTA, 7 Bacterial culture 4 spiked with 40 µg/L OTA.

In the present study, bacterial strains isolated from the Korean fermented food Kimchi were tested for their OTA reducing abilities. Bacterial culture broth of each isolated strain was spiked with 40 µg/L of OTA, followed by incubation and subsequent OTA extraction. The isolated extracts were analyzed by TLC. While bacterial strains unable to reduce OTA were visualized as bluish-green fluorescent spots on TLC plates (lanes 4, 6, and 7 in Fig. 2), those with OTA-reducing ability were showed no fluorescent spots (lane 5 in Fig. 2).

The bacterial strain KU-153, possessing OTA-reducing abilities, was identified as *B. subtilis* based on 16 S rRNA gene sequence analysis as described previously^28^. According to BLAST analysis, the gene sequence of the isolated strain showed 100% similarity with other *B. subtilis* strains in GenBank, confirming its identity as *B. subtilis*. Previous reports have also confirmed the degradation and adsorption of mycotoxin in culture medium by *B. subtilis*^29^. In addition, Shi *et al*.^30^ isolated a *B. subtilis* strain from fresh elk droppings and found that it could prevent OTA contamination and degrade OTA in crops. In addition, Petchkongkaew *et al*.^31^ isolated a *Bacillus* strain from a Thai fermented soybean food product and showed that it was able to inhibit the growth of an OTA-producing fungus.

### Growth kinetics and OTA reduction ability of *B. subtilis*

**Figure 3:**
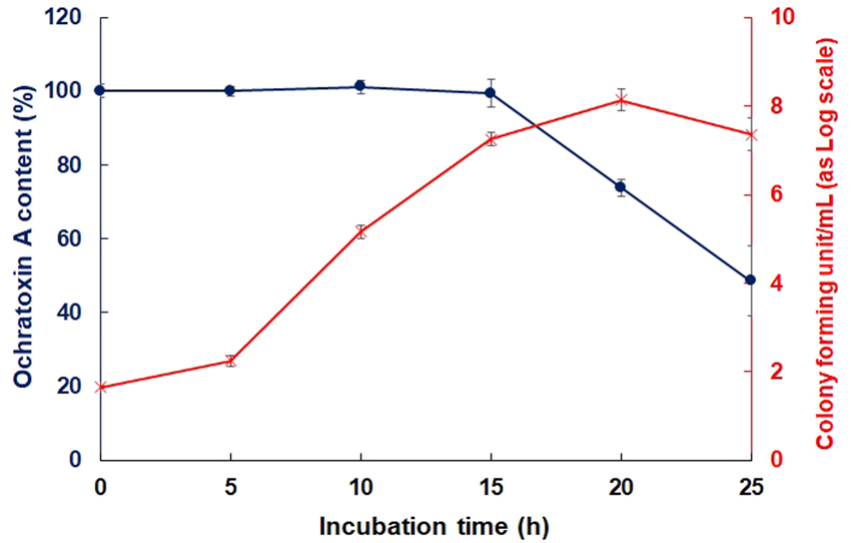
Relationship between *Bacillus subtilis* growth rate and ochratoxin A reduction levels in culture media.

The kinetics of OTA reduction by *B. subtilis* KU-153 were examined in culture medium. Monitoring of the bacterial cell counts and OTA content showed that an increase in the bacterial population led to a reduction in OTA content. In the presence of *B. subtilis*, the OTA content was reduced by 58.10% after 25 h (Fig. 3). The colony forming unit (cfu of the bacterial growth medium increased up to 20 h, after that, it reached a plateau (log CFU 8.12 to 7.25 after 20 h), confirming the stationary phase of bacterial growth (15–20 h). Concurrently, the OTA content was also reduced by 20.01% and 51.35% at 15 and 20 h, respectively (Fig. 3). Similar results have been reported for other *Bacillus* strains able to degrade aflatoxins, one of the major groups of mycotoxins^29^. Fuchs *et al*.^32^ also showed that the adsorption of mycotoxins is correlated with the amount of bacteria in the culture reaction mixture, and significant OTA reductions were seen, particularly when the bacterial population in the culture medium was approximately 10^8^ CFU/mL.

In addition, Peteri *et al*.^33^ and Piotrowska^16^, who studied the ability of *Lactobacillus* species and yeast strains to reduce OTA, reported reductions in OTA in culture media and food products in the range of 8–28%. Controversially, Mateo *et al*.^34^ reported that several strains of *Oenococcus oeni* reduced the OTA content in culture medium by 50–70%, while Fuchs *et al*.^32^ reported that *Lactobacillus acidophilus* reduced OTA in broth medium by>98%. Petchkongkaew *et al*.^31^ reported that a *Bacillus* strain isolated from a fermented soybean degraded OTA by 92.5%. Further, Kapetanakou *et al*.^21^ confirmed in their study that all tested yeast composites resulted in higher OTA reductions (65%) in culture media and beverages than bacterial species (2–25%) including species of *Bacillus* and *Lactobacillus*. Such discrepancies in OTA reduction results from different reports may be associated with the diversity of strains and differences in evolutionary origin, suggesting that detection and selection of OTA-reducing strains remains challenging. The results obtained by several researchers have shown that OTA is removed or reduced from the medium in the absence of metabolically active cells, suggesting that the OTA may be adsorbed by the cells. In other words, as the number of *B. subtilis* cells increased in the reaction suspension, more OTA was removed from the medium, confirming the direct relationship between cell number and OTA reduction^16,35,36^.

### Assay validation

In this study, validation of the analytical method showed good linearity. The linear regression coefficient of the standard solution curve (*y* = 455.11*x* + 50.854) for OTA within the concentration range of 0–50 µg/L was 0.9994. The mean recovery level of OTA was 49.53 ± 3.11 while using 50 µg/L as the injected concentration (Table 1). The LOD values obtained were 2.24 µg/L and 8.32 µg/L, respectively. The results of the relative standard deviation (RSD) indicated that the method was compatible, as it showed good precision with an RSD < 20% (Table 1).

**Table 1:** Assay validation for analysis of ochratoxin A using HPLC.

Standard ochratoxin A concentration (µg/L)	Recovered ochratoxin A concentration(µg/L)	RSD (%)	LOD (µg/L)	LOQ (µg/L)
5	4.92 ± 0.75	15.22	2.24	8.32
10	9.81 ± 0.83	8.48		
30	30.86 ± 2.17	7.02		
50	49.53 ± 3.11	6.28		

LOD: Limit of detection; LOQ: Limit of quantification.

### Comparison of OTA reduction abilities of extracellular fraction, intracellular fraction, and cells of *B. subtilis*

**Figure 4:**
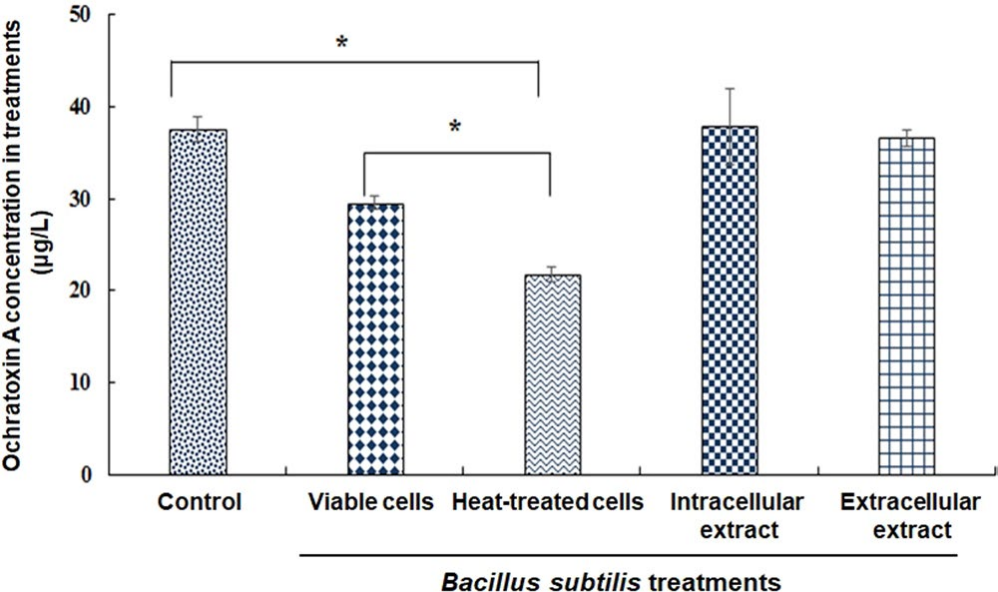
Ochratoxin A reduction after 24 h treatment with viable and heat-treated *Bacillus subtilis*, intracellular extract, and extracellular extracts of *Bacillus subtilis*. *^,^**^,^***Significantly different with each other at *p* < 0.05 by Duncan’s multiple range test.

**Figure 5:**
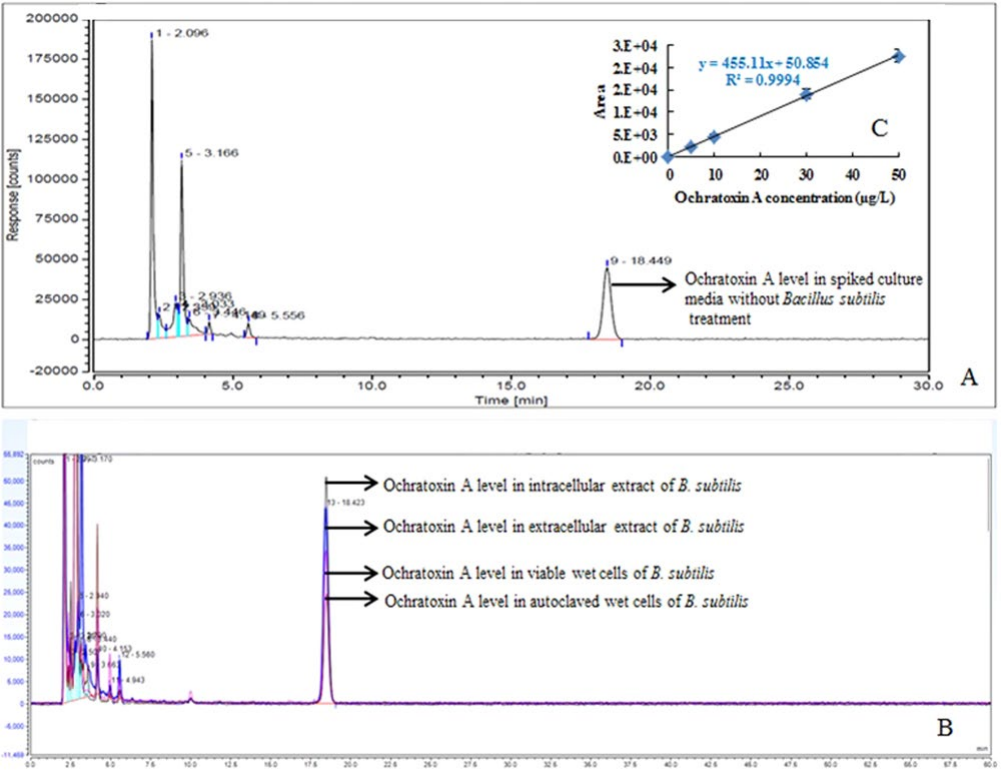
HPLC chromatographs for ochratoxin A levels. (**A**) OTA level in spiked culture media without *Bacillus subtilis*, (**B**) ochratoxin A level in spiked culture media treated with intracellular extract, extracellular extract and viable cells and heat-treated cells of *Bacillus subtilis*, (**C**) standard curve of ochratoxin A with different concentrations.

To further elucidate the basis for the OTA-reducing ability of the *B. subtilis* strain, we compared the OTA reduction abilities of the intracellular and extracellular fractions and of viable and heat-treated cells of *B. subtilis*. The results revealed no reduction in OTA content by intracellular and extracellular fractions. However, both viable and heat-treated *B. subtilis* cells showed significant (*p* < 0.05) reductions in OTA levels (Figs 4 and 5) with heat-treated *B. subtilis* cells showing a greater OTA reduction ability (45%) than viable cells (22%) (Fig. 4). Similarly, Pietri *et al*.^33^ observed the OTA eliminating capacity of *O. oeni* during the exponential growth phase and in cell-free extracts and found that the amount of OTA was reduced during bacterial growth by 10.99–28.09%, whereas no OTA elimination was observed using cell-free extracts. Turbic *et al*.^37^ also reported that only viable and non-viable cells of *Lactobacillus rhamnosus* reduced OTA in food samples by 20%. Fiori *et al*.^38^ reported that autoclaved yeast cells exhibited high biocontrol efficiencies against OTA in grape juice.

Fuchs *et al*.^32^ reported that the viability of lactic acid bacteria cells plays an important role in OTA reduction, since the heat-inactivated cells of lactic acid bacteria were found to result in only a moderate reduction in OTA of 11%. The results observed by Fuchs *et al*.^32^ differed therefore from our results and from others reported in the literature. For example, it was reported that in the case of mycotoxins, such as aflatoxin B1 and zearalenone, heat-inactivated bacteria bind these toxins equally well or even more effectively than viable cells do^39–41^. In addition, Piotrowska^16^ reported results consistent with those of our study, finding that heat-inactivated *Lactobacillus* cells reduced OTA more efficiently than live cells did. This supports the idea that the majority of OTA is not reduced by bacterial cells in the active growth phase. Higher OTA adsorption by heat-killed rather than live cells may be explained by changes occurring in the bacterial cell wall induced by high temperature. In other words, protein denaturation and pore generation leading to increased permeability of the external layers of the cell wall result in a greater number of active sites responsible for the absorption of different compounds^42^. As reported previously, a crucial role in the binding of OTA by bacterial biomass is played by cell wall components, such as peptidoglycan and polysaccharides, as well as teichoic and lipoteichoic acids^43^. Another factor responsible for OTA removal may be the hydrophobic nature of the bacterial cell wall^16^. In this study, heat-treated cells were more effective in terms of OTA removal, indicating that the hydrophobic surface of the thermally inactivated cells is responsible for this process. The fact that OTA is also bound by live lactic acid bacteria, despite the hydrophilic nature of their surface, is probably attributable to the presence of so-called hydrophobic pockets on the surface^43^. Although the surface of other bacterial species such as *Escherichia coli* also exhibit similar characteristics, *E. coli* has not been observed to bind OTA. This suggests that OTA adsorption is also affected by other factors, such as the chemical composition of the cell wall, which contains more lipopolysaccharides in gram-negative bacteria.

Tinyiro *et al*.^27^ reported the adsorption and degradation of zearalenone by *Bacillus natto* and observed that the amounts of zearalenone bound by viable, autoclaved, and acid-treated *B. natto* cells were 89.5%, 73.5%, and 70.5%. Although acid-treated cells bound less zearalenone than viable or autoclaved cells, this was not statistically significant (*p* < 0.05), suggesting the initial point of the presence of zearalenone to a lesser extent of protein binding sites^27,41^. Research evidence has also shown that sub-lethal high-pressure homogenization treatment affects the membrane fatty-acid desaturase enzymes that are involved in the active response of bacterial cells to high-pressure stress^44^. In addition, high-pressure homogenization treatment has been reported to alter the activity of several microbial enzymes as well as those that naturally occur in food matrices^45,46^. Heat treatment has been proposed to broaden the antimicrobial spectrum of lysozymes against gram-negative bacteria and increase activity against gram-positive ones^47^. In addition, higher OTA adsorption by dead rather than live cells may be explained by changes occurring in the bacterial cell wall induced by high temperature, that is, protein denaturation and pore generation leading to increased permeability of the external layers of the cell wall. This in turn results in a greater number of active sites responsible for the adsorption of different compounds^48^.

The adsorption of bacteria by dead biomass is highly beneficial and may be used in practice, as this decontamination method facilitates the preservation of the organoleptic properties of the products. Bacterial cells may thus be used as dietary supplements, preventing the absorption of toxins in the human gastrointestinal tract. According to Tuomola *et al*.^49^, high temperatures decrease the adhesion of bacteria to the intestinal mucosa, together with the toxins adsorbed on the bacteria. It has been suggested that adhesion of bacteria to the mucosa decreases with the degree of denaturation of the proteins responsible for the adhesion process, while the hydrocarbons binding OTA were not degraded^50^.

### OTA reduction ability of *B. subtilis* cells in wine

**Figure 6:**
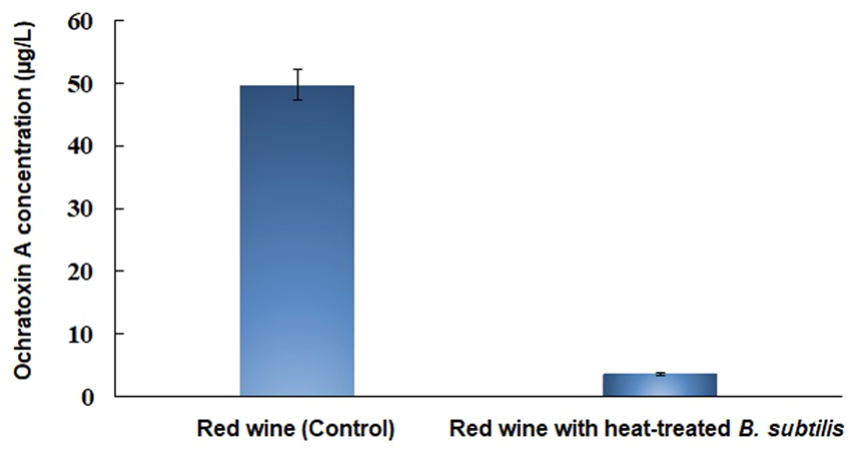
Ochratoxin A reduction ability of *B. subtilis* strains in red wine.

Global research findings have shown that wine is an important beverage in worldwide trade. The rate of contamination of wine also varies based on the raw materials and origin of the grapes. In Italy, a variety of wines have been extensively surveyed and found to exhibit high rates of OTA contamination, especially in red wines (78.4%), followed by rose and white wines. Therefore, in the present study, wine samples were used to confirm the practical applicability of using heat-treated cells of *B. subtilis* to reduce OTA contents. As a result, we observed that the amount of OTA artificially added to the red wine sample was reduced 3.63 µg/L (~90%) when treated with heat-treated cells of *B. subtilis* (Fig. 6). Based on this, the ability of *B. subtilis* heat-treated cells to reduce OTA may be promising, as it may also allow the biological elimination of other mycotoxins in the food matrix in a similar manner. Since these bacteria provide a source of bioactive components, they could be used for detoxification of various food products contaminated with OTA.

As to resolve the question of safety issue toward the consumer who drink the wine containing *Bacillus* cells attached with mycotoxin such as OTA. In earlier research, Vinderola and Ritieni^51^ demonstrated that binding of mycotoxin with probiotic bacterial cell is a property that can be retained after gastric digestion. Certain probiotic strains can bind and remove mycotoxins from liquid media. Normally, eukaryotic cell cultures show that the complex probiotic-mycotoxin is less adhesive to enterocytes than the probiotic alone, thus favoring the elimination of this complex from the gut through feces. These approaches confirm the safer use of our strategy to reduce OTA in wine. In addition, this work is the first part of our research, therefore, in the present work, we have confirmed OTA binding efficiency in selected *B. subtilis* strain which was isolated from Kimchi. Since by using this strategy, it is not possible to remove *Bacillus* cells bounded with OTA on its surface, therefore in our further experimental steps, we planned to use heat-treated *B. subtilis* cells immobilized in edible polymeric materials in the form of beads as an effective tool to reduce OTA contents from liquid matrix-based food products (data not shown).

### Mechanistic hypothesis

We next speculated on the mechanism by which our isolate, *B. subtilis* KU-153, was able to reduce the OTA content. A number of similar studies to visualize the effect of heat-inactivated bacterial cells on OTA reduction have been conducted^52^. Although some gram-positive bacteria such as *Bacillus* species, *Lactobacillus* species, *Bifidobacterium* species, and *Streptococcus thermophilus* are known to possess OTA adsorption abilities^16,30–32^, OTA adsorption by gram-negative bacteria has not been reported so far. While the OTA adsorption mechanism by gram-positive bacteria has not yet been fully demonstrated, it is supposed that physical and chemical characteristics of the cell wall, i.e., the chemical composition of the cell wall, thickness of the peptidoglycan, absence of an outer membrane barrier, and environmental conditions, play an important role in OTA adsorption^16,43,48,53–55^.

Previously, Niderkorn *et al*.^55^ stated that the peptidoglycan of *B. subtilis* can adsorb fumonisin, a mycotoxin, and that the specific amino acid sequence of the peptide bridges between *N*-acetylmuramic acid chains in peptidoglycan is particularly important in the efficiency of OTA adsorption. In addition to the specific amino acid sequence, environmental conditions such as pH might also affect OTA adsorption by some lactic acid bacteria^55,56^. Depending on the environmental pH, OTA adsorption by the cell wall varies, with the highest OTA adsorption activity observed at pH 3.0^57^. Similarly, Fuchs *et al*.^32^ also reported that the OTA adsorption activity by viable lactic acid bacteria tended to increase with decreasing external pH in the range of pH 5–8. Similarly, in our study, we found that the OTA adsorption activity by *B. subtilis* tended to increase with decreasing external pH in the range of pH 3–7 (data not shown). In addition, our results also revealed that *B. subtilis* cells treated at 121 °C for 15 min showed higher OTA adsorption activity than non-treated *B. subtilis* cells. Thus, based on the above results, we hypothesize that the higher adsorption activity in heat-treated cells could be the result of new binding sites for OTA created by the heat exposure of the cell membrane and partial breakdown of peptidoglycan after heat treatment of *B. subtilis* cells^42^. However, further studies are necessary to elucidate the exact mechanism by which *B. subtilis* is able to adsorb OTA.

**Figure 7:**
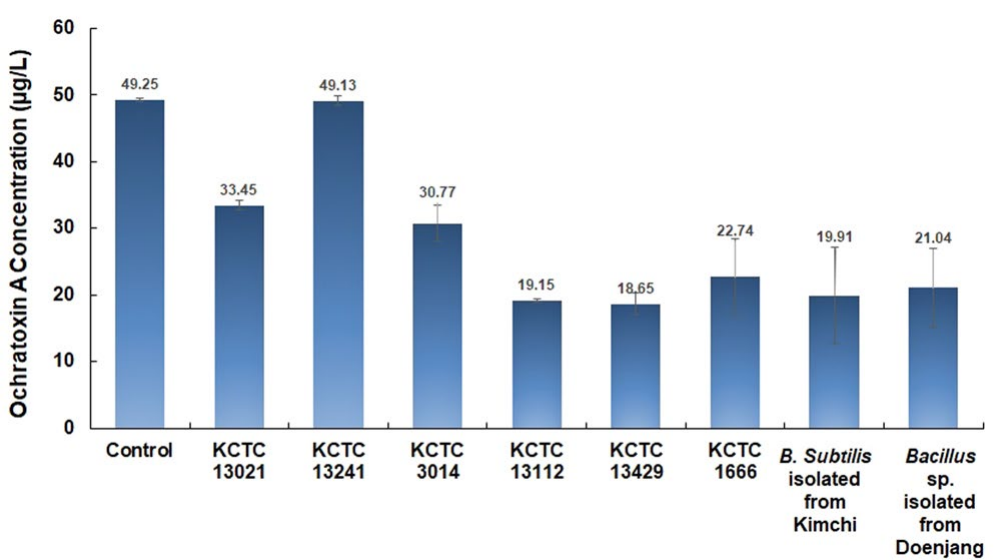
Applicability of heat-treated *Bacillus subtilis* cells for ochratoxin A reduction ability in red wine samples.

In order to compare the OTA reduction potential of our *B. subtilis* KU-153 strain with that of other *B. subtilis* strains, *B. subtilis* KCTC 13021, KCTC 13241, KCTC 3014, KCTC 13112, KCTC 13429, KCTC 1666, and *B. subtilis* isolated from a Doenjang sample were tested as reference strains. The results showed that our *B. subtilis* strain isolated from Kimchi showed potential for OTA reduction along with some other *B. subtilis* strains, including *B. subtilis* isolated from Doenjang, *B. subtilis* KCTC 13429, and *B. subtilis* KCTC 13112. In contrast, other *B. subtilis* strains such as KCTC 13021, KCTC 13241, KCTC 3014, and KCTC 1666 showed low OTA reduction abilities (Fig. 7). As in results, it does not appear that all strains of *B. subtilis* possess the ability to reduce OTA levels, though some of the tester strains (KCTC 13429 and KCTC 13112) did, though not necessarily at the same bioactive level as the strain was isolated from Kimchi (KU-153). Therefore, the results emphasize that KU-153 could also be used as an alternate over Doenjang isolated *B. subtilis*, KCTC 13429 and KCTC 13112 for its OTA reduction ability.

This study elucidated the process of OTA reduction by a strain of *B. subtilis* isolated from the Korean traditional fermented food Kimchi. We observed that levels of spiked OTA were significantly reduced in medium with heat-treated *B. subtilis* cells. These findings reinforce the growing body of evidence that *B. subtilis* isolated from Kimchi has excellent potential for use as a biocontrol agent against OTA contamination in various foods and/or agricultural products. However, greater numbers of *B. subtilis* cells are required in pilot scale which is not feasible industrially and was considered as a limiting factor of this study. As a concluding remark, further studies are in progress to make efficient use of *B. subtilis* cells in cost-effective industrial applications for food and feed products.

Furthermore, if heat-treated strains of *B. subtilis* can immobilize OTA and reduce levels of this mycotoxin in food and feed prior to and after ingestion, it would be necessary to demonstrate that OTA cannot be inadvertently be released from bacterial cell walls during the normal digestive process by humans or animals or the action of another microorganism as part of the normal microbiota of the digestive tract. Fortunately, the preliminary data from this and other studies do not indicate that this is so based on the current evidence.
